# Clinical phenotype and laboratory characteristics of 93 patients with congenital fibrinogen disorders from unrelated 36 families

**DOI:** 10.1016/j.rpth.2024.102445

**Published:** 2024-05-17

**Authors:** Dandan Tian, Juan Liang, Hui Gao, Xiaojun Xu, Wenjian Nie, Mingwei Yin, Jintu Lou, Hong-Qiang Shen

**Affiliations:** 1 Department of Clinical Laboratory, Children’s Hospital, Zhejiang University School of Medicine, National Clinical Research Center for Child Health, Hangzhou, China; 2 Department of Hematology-Oncology, Children’s Hospital, Zhejiang University School of Medicine, National Clinical Research Center for Child Health, Hangzhou, China; 3 Department of Blood Transfusion, Children’s Hospital, Zhejiang University School of Medicine, National Clinical Research Center for Child Health, Hangzhou, China

**Keywords:** bleeding, congenital fibrinogen disorders (CFDs), dysfibrinogenemia, hypodysfibrinogenemia, hypofibrinogenemia

## Abstract

**Background:**

Congenital fibrinogen disorders (CFDs) are rare bleeding disorders (RBDs) caused by mutations in 1 of the 3 fibrinogen genes (*FGA*, *FGB*, and *FGG*).

**Objectives:**

To investigate the clinical phenotype, laboratory features, diagnosis, treatment, and prognosis of CFDs.

**Methods:**

Clinical data of 93 subjects with CFDs identified from June 2018 to December 2023 were retrospectively analyzed.

**Results:**

Among the 93 patients, there were 46 males (49.5%) and 47 females (50.5%), with a median age of 23 years. Fifty-three of 93 (57%) subjects experienced bleeding, 3/93 (3.2%) experienced thrombosis, and 37/93 (39.8%) were asymptomatic. Females were more prone to experience bleeding (*P* < .0001). The 93 patients exhibited prolonged thrombin time, significantly decreased fibrinogen activity (Fg:C), and normal or decreased fibrinogen antigen. The 93 patients included 3 with hypofibrinogenemia, 16 with hypodysfibrinogenemia, and 74 with dysfibrinogenemia. Among the 53 patients with bleeding, bleeding episodes were identified in 3.8% (2/53), 20.8% (11/53), and 75.5% (40/53) patients with hypofibrinogenemia, hypodysfibrinogenemia, and dysfibrinogenemia, respectively. Genetic analysis was performed on 22 cases from 8 pedigrees, revealing 10 mutations, including 1 novel splice mutation. Twenty-eight (30.1%) subjects received replacement therapy to treat or prevent bleeding, consisting of 8 fresh frozen plasma transfusions, 3 packing and suture treatment, and 61 fibrinogen infusions.

**Conclusion:**

Most patients with CFDs have mild or no bleeding symptoms. Fg:C combined with fibrinogen antigen and pedigree investigation can improve the feasibility and accuracy of diagnosis of CFDs. The severity of bleeding symptoms was negatively correlated with Fg:C.

## Essentials

• Congenital fibrinogen disorders (CFDs) are rare hereditary bleeding diseases.
• We analyzed the clinical data of 93 patients with CFDs from 36 families.
• Most patients with CFDs have mild or no bleeding symptoms.
• The severity of bleeding was negatively correlated with fibrinogen activity levels.

## 1 Introduction

Fibrinogen is a 340-kDa plasma glycoprotein composed of 2 sets of symmetrical trimers, each formed by the Aα, Bβ, and γ chains. These chains are encoded by the *FGA*, *FGB*, and *FGG* genes, respectively [1]. Fibrinogen deficiencies include quantitative deficiencies (afibrinogenemia and hypofibrinogenemia) and qualitative deficiencies (dysfibrinogenemia and hypodysfibrinogenemia) of fibrinogen [2]. Congenital fibrinogen disorders (CFDs) are rare bleeding disorders (RBDs) caused by mutations in 1 of the 3 fibrinogen genes (*FGA*, *FGB*, and *FGG*) [3,4]. However, recent data from the World Hemophilia Federation survey indicate that CFDs account for 8% of all RBDs, and their incidence is increasing compared with other autosomal heritable RBDs [5,6]. Previous studies on CFDs mainly consisted of case reports or limited family reports, making it difficult to establish correlations between laboratory features, clinical phenotype, treatment, and prognosis due to their rarity.

Herein, we performed a retrospectively analysis of 93 subjects from 36 unrelated families with CFDs to explore the clinical phenotype, laboratory features, treatment, and prognosis of CFDs.

## 2 Methods

### 2.1 Identification of study subjects and clinical evaluation

We recruited child probands with reduced fibrinogen activity (Fg:C) and prolonged thrombin time (TT) without adequate clinical explanation. Those child probands were patients from the Children’s Hospital of Zhejiang University, School of Medicine (Hangzhou, China), between June 2018 and December 2023. We identified potential subjects including child probands and their family members through pedigree investigation. We determined if they fulfill the following inclusion criteria: 1) decreased Fg:C, 2) normal or prolonged TT, and 3) normal liver and kidney functions. The cutoff thresholds used to define “normal,” “prolonged,” and “reduced” test results were the 95% reference intervals for each test derived at each laboratory. Those with identifiable causes of acquired CFDs [7] and those family members with normal coagulation function were excluded.

All potential subjects underwent clinical evaluation after obtaining written informed consent. We recorded the historical clinical symptoms of bleeding or thrombosis by inpatient/outpatient medical history, patient interview, and telephone questionnaire. The clinical information of bleeding includes the presence or absence of bleeding symptoms, type of bleeding, bleeding frequency, quantity of bleeding, and the level of medical attention and treatment required for each. Clinical bleeding manifestations were quantified using the consensus International Society on Thrombosis and Haemostasis bleeding assessment tool (ISTH-BAT) [8,9]. All procedures were conducted in accordance with the Declaration of Helsinki, and the study was approved by the Ethics Committee of Zhejiang University School of Medicine with the ethics board number 2023-IRB-0189-P-01.

### 2.2 Coagulation tests

Venous blood samples were collected in tubes with 0.109 mol/L sodium citrate. All blood samples were centrifuged at 1500 *g* for 15 minutes to produce platelet-poor plasma. The prothrombin time (PT), activated partial thromboplastin time (aPTT), and TT were detected by the 1-stage clotting method. The Fg:C and fibrinogen antigen (Fg:Ag) were tested by the Clauss [10] and PT-derived methods, respectively. PT-derived method is a clinical practical method and correlates well with immunologic assays, the gold standard measure of Fg:Ag [[11], [12], [13]]. In the study, the normal reference values for all coagulation tests in children were as follows: PT, 9.0 to 14.0 seconds; APTT, 23.0 to 38.0 seconds; TT, 15.0 to 22.0 seconds; and Fg, 1.8 to 4.0 g/L); the normal reference values in adults were as follows: PT, 9.0 to 14.0 seconds; APTT, 22.0 to 35.0 seconds; TT, 14.0 to 21.0 seconds; and Fg, 2.0 to 4.0 g/L. All these assays were performed by CS-5100 automated coagulation analyzer (Sysmex Corporation). All operations were conducted following the manufacturer’s protocols.

### 2.3 The classification of CFDs

According to the recommendation [5,[14], [15], [16]] of domestic and foreign experts on the diagnosis and classification of CFDs, CFDs include afibrinogenemia (complete absence of fibrinogen), hypofibrinogenemia (Hypo-; proportional decrease of functional and antigenic fibrinogen levels), dysfibrinogenemia (Dys-; decreased functional and normal antigenic fibrinogen levels), and hypodysfibrinogenemia (Hypodys-; discrepant decrease of functional and antigenic fibrinogen levels).

### 2.4 DNA sequence analysis

DNA specimens for gene analysis were extracted from the anticoagulant blood samples using DNA Extraction Kit (Tiangen) according to the manufacturer’s protocol. The primer sequences were designed according to the Fg gene sequence from the GenBank (*FGA*:NM_021871, *FGB*:NM_005141, and *FGG*:NM_021870), covering all exon regions of *Fg* gene and its flanks sequence. All the polymerase chain reaction (PCR) amplifications were carried out in a thermal circulator (Eppendorf). A total volume of PCR system was 25 μL, including 2×Es Taq MasterMix of 12.5 μL, the forward and reverse primers of 1 μL, respectively, ddH_2_O of 8 μL, and the DNA template of 2.5 μL. The amplification of PCR products was conducted under the following conditions: 5 minutes at 95 °C, 35 cycles for 40 seconds at 95 °C, 50 seconds at 55 °C, 1 minute at 72 °C, and 10 minutes at 72 °C. The purified PCR products were sent directly to Kindstar Global for sequencing, and the results were analyzed by Chromas software and NCBI GenBank.

### 2.5 Statistical analysis

Normally distributed variables are presented as mean ± SD, while nonnormally distributed variables are presented as medians and IQR (25th-75th percentiles). Normally distributed variables were analyzed by independent sample *t* test, while nonnormally distributed variables were analyzed by the nonparametric Mann–Whitney U-test. Categorical variables were compared with use of chi-squared analysis. The relationships between 2 variables were analyzed using the Spearman correlation analysis. Data were analyzed using SPSS 20.0 (SPSS), and a 2-sided *P* value of <.05 was considered statistically significant.

## 3 Results

### 3.1 Demographic and clinical characteristics

We included 93 subjects in the study, with 46 males and 47 females, with a median age of 23 years (range, 0.33-68 years). Among them, there were 37 child probands (2 probands are siblings) and 56 relatives from 36 unrelated families. Twenty-two (59.5%) child probands were first identified at the preoperative clotting screening, which included cases such as excision of skin abscess (*n* = 1), frenectomy (*n* = 1), aspiration of hematoma of scalp (*n* = 1), excision of hydrocele of testis (*n* = 1), indirect inguinal hernia hernioplasty (*n* = 1), hemangiomatectomy of lower limb (*n* = 1), adenotonsillectomy (*n* = 2), eyelid mass excision (*n* = 3), and circumcision (*n* = 11). Four (10.8%) child probands were identified during the physical examination (*n* = 4), and 2 (5.4%) probands were identified in inpatient care (abdominal pain, *n* = 1; pneumonia, *n* = 1). Furthermore, 9 (24.3%) child probands were first identified during the investigation of bleeding, including cases of spontaneous epistaxis (*n* = 7), bruise (*n* = 1), and bleeding from minor wounds (*n* = 1; Supplementary Table S1).

**Figure 1:**
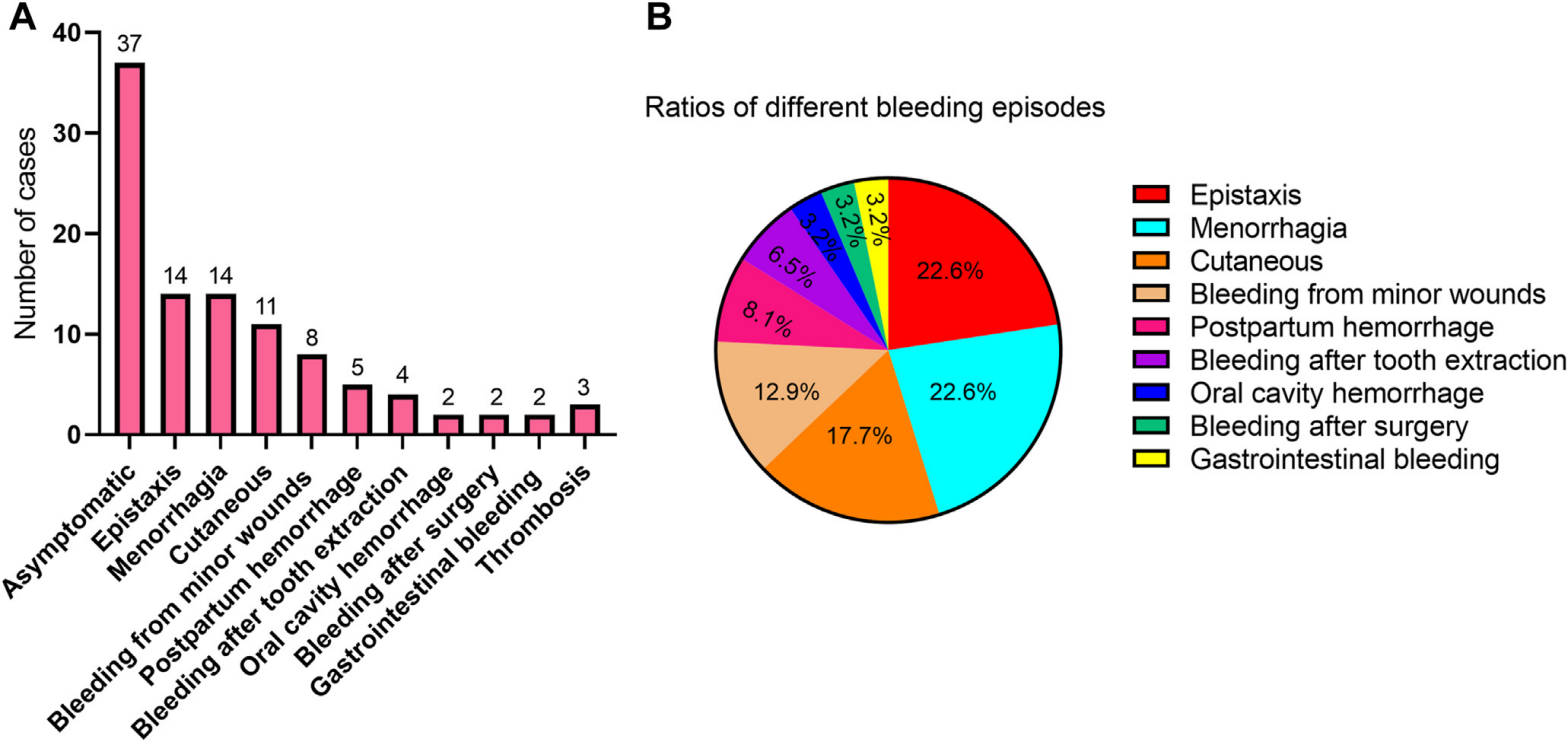
Clinical characteristics of the 93 study subjects with congenital fibrinogen disorders. (A) Number of cases of different clinical symptoms in patients with congenital fibrinogen disorders. The number at the top of the bar chart refers to the number of cases of different bleeding episodes. (B) Ratios of different bleeding episodes in the total of 62 bleeding episodes of 53 patients.

Of the 93 patients, 37 (39.8%) were asymptomatic, while 53 (57%) had bleeding symptoms and 3 (3.2%) had suffered from thrombotic episodes (Table 1). A total of 62 bleeding episodes occurred in 53 patients, with a median bleeding score of 2 (range, 0-7). The most common bleeding symptom was spontaneous minor-to-moderate epistaxis (22.6%) and menorrhagia (22.6%), followed by bruise (17.7%), bleeding from minor wounds (12.9%), postpartum hemorrhage (8.1%), bleeding after tooth extraction (6.5%), gastrointestinal bleeding (3.2%), gum bleeding (3.2%), and bleeding after surgery (3.2%; Figure 1A, B). The ISTH-BAT bleeding score of >1 was identified in only 36/93 (38.7%) subjects, with a median bleeding score of 3 (range, 2-7). There were 6, 2, and 13 patients with ISTH bleeding scores of ≥4 in males, ≥6 in females, and ≥3 in children, respectively.

**Table 1:** Characteristics of study participants (*N* = 93).

Parameters	Total population (*n* = 93)	Those with symptoms (*n* = 56)	Those without symptoms (*n* = 37)	*P* value
Race/ethnicity				
Asian	93 (100)	56 (100)	37 (100)	
Age (y), median (IQR)	23 (8.21-37.5)	30.5 (8.1-38)	11 (8-35)	.20
Sex (male/female)	46/47	22/34	24/13	.02^a^
Bleeding episodes, *n* (%)	53 (57)	53 (94.6)	0	<.0001^b^
Thrombosis, *n* (%)	3 (3.2)	3 (5.4)	0	.27
ISTH-BAT bleeding score, median (IQR)	1 (0-3)	2 (1-3)	0 (0-0)	<.0001^b^
Laboratory characteristics (mean ± SD)				
PT (s)	12.65 ± 0.95	12.65 ± 1.02	12.65 ± 0.86	.98
aPTT (s)	28.35 ± 2.58	28.39 ± 2.60	28.31 ± 2.59	.88
TT (s)	29.17 ± 4.09	29.39 ± 2.63	28.84 ± 3.33	.53
Fg:C (g/L)	0.45 ± 0.23	0.43 ± 0.22	0.46 ± 0.25	.54
Fg:Ag (g/L)	2.08 ± 0.44	2.08 ± 0.47	2.08 ± 0.39	.89

aPTT, activated partial thromboplastin time; Fg:Ag, fibrinogen antigen; Fg:C, fibrinogen activity; ISTH-BAT, International Society on Thrombosis and Haemostasis bleeding assessment tool; PT, prothrombin time; TT, thrombin time.

a *P* < .05 (with symptom vs without symptom groups).

b *P* < .0001 (with symptom vs without symptom groups).

**Figure 2:**
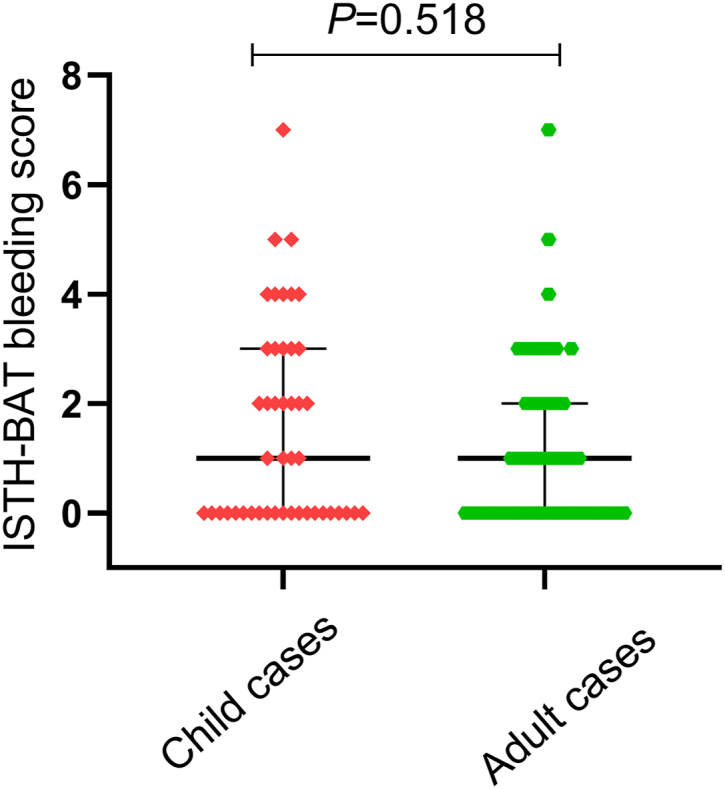
Distribution of bleeding scores of 93 patients with congenital fibrinogen disorders by age. Bleeding scores (median, IQR) in subjects were determined using the ISTH bleeding assessment tool and were compared between the child and adult groups. ISTH-BAT, International Society on Thrombosis and Haemostasis bleeding assessment tool.

**Figure 3:**
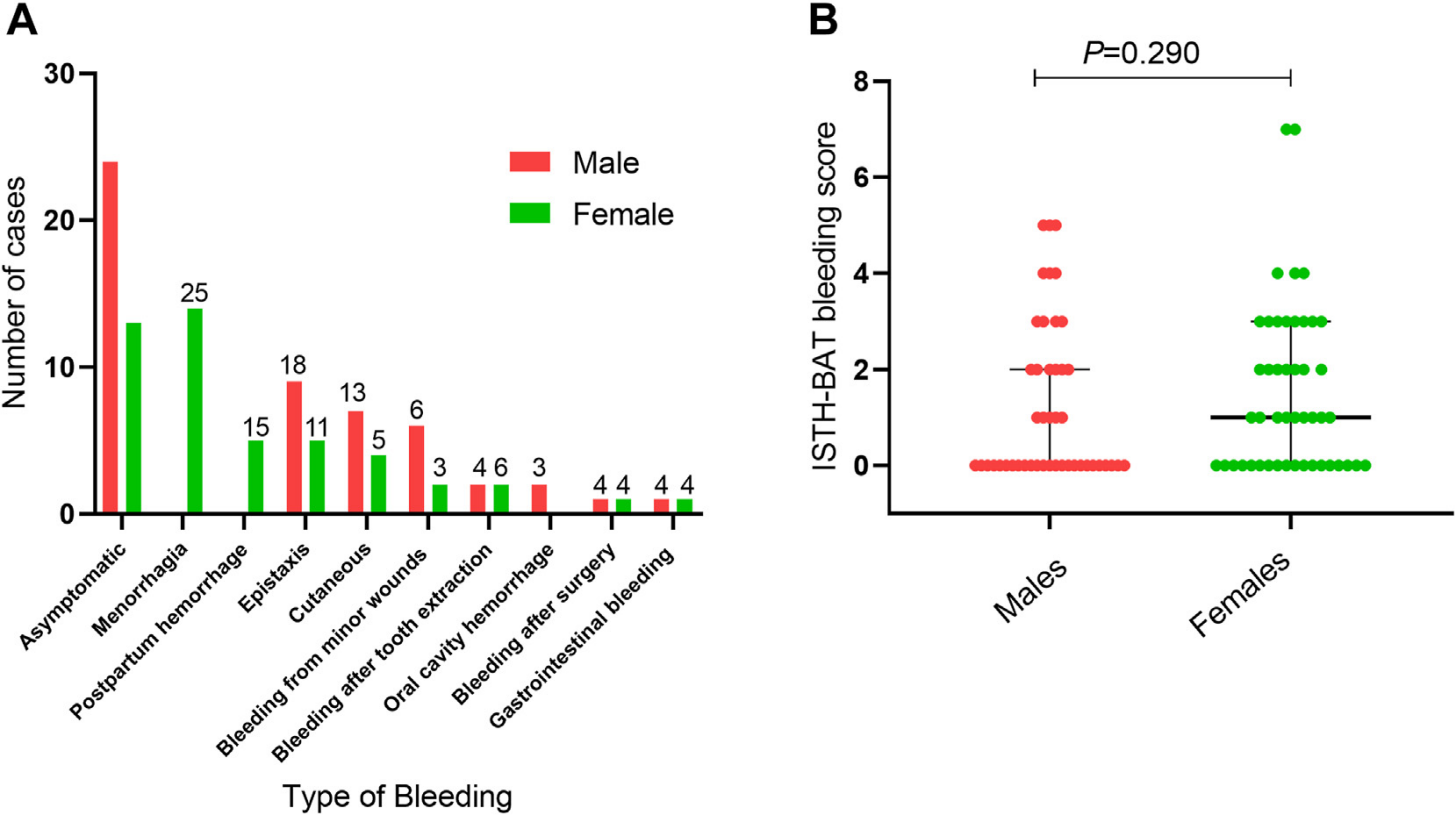
Distribution of bleeding episodes of 93 patients with congenital fibrinogen disorders by sex. (A) Number of cases of bleeding episodes at different anatomical sites between female and male groups. The number at the top of the bar chart refers to the total scores for different bleeding episodes. (B) Bleeding scores (median, IQR) were compared between the male and female groups.

All 93 subjects included 45 children (28 males, 17 females) and 48 adults (18 males, 30 females). The incidence of bleeding was slightly higher in adults (30.1%) than in children (26.9%), whereas the overall ISTH-BAT bleeding scores in children (median, 1; range, 0-7) were similar to those in adults (median, 1; range, 0-7; *P* > .05; Figure 2). Furthermore, a higher percentage of females (33.3%) experienced bleeding compared with males (23.7%; *P* < .0001; Figure 3A). The overall ISTH-BAT bleeding scores in females were slightly higher (median, 1; range, 0-7) than those in males (median, 0; range, 0-5; *P* > .05; Figure 3B).

The 30 women had 43 pregnancies, including 30 full-term vaginal births, 11 cesarean deliveries, and 2 early spontaneous abortions. The complete pregnancy medical history of 17 women was obtained. Noticeably, 1 patient had 2 consecutive abortions in the 8th and 11th week of pregnancy (autoimmune deficiency–induced miscarriage was excluded). There were 5 cases of postpartum hemorrhage, including 2 cases of vaginal delivery and 3 cases of cesarean delivery. Furthermore, 3 women had suffered from postpartum deep venous thrombosis (DVT), including 2 sisters (aged 36 and 38 years, family 10; Supplementary Table S1) and 1 36-year-old patient (family 36, Supplementary Table S1). DVT was confirmed by positive ultrasonography (thrombosis in femoral and/or popliteal veins), and the common causes of acquired (long postpartum bed rest) and inherited thrombophilia [17] have been ruled out.

### 3.2 Laboratory characteristics of CFDs

**Figure 4:**
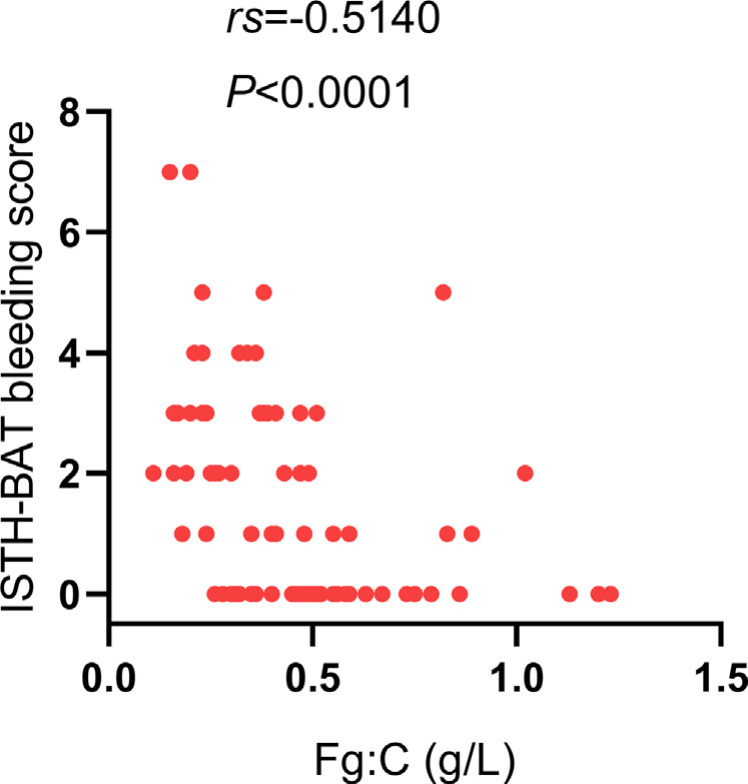
Correlation analysis of fibrinogen activity (Fg:C) with the ISTH-BAT bleeding score. ISTH-BAT, International Society on Thrombosis and Haemostasis bleeding assessment tool.

All 93 subjects exhibited normal or slightly prolonged PT (mean, 12.6 seconds; range, 10.4-16.0 seconds), normal aPTT (mean, 28.4 seconds; range, 21.0-36.2 seconds), significantly prolonged TT (mean, 29.2 seconds; range, 23.6-46.4 seconds), significantly decreased Fg:C (mean, 0.45 g/L; range, 0.11-1.23 g/L), and normal or decreased Fg:Ag (mean, 2.08 g/L; range, 1.27-3.53 g/L). There was no significant difference in PT, aPTT, TT, Fg:C, and Fg:Ag between patients with and without symptoms (*P* > .05; Table 1). Furthermore, the bleeding score was negatively correlated with Fg:C levels (*rs* = −0.5140, *P* < .0001; Figure 4).

For women, Fg:C levels were slightly lower in patients with menorrhagia (0.42 ± 0.04 g/L), 2 abortions (0.44 ± 0.04 g/L), and postpartum hemorrhage (0.33 ± 0.14 g/L) than in those without menorrhagia (0.46 ± 0.21 g/L, *P* = .60), abortion (0.47 ± 0.24 g/L, *P* = .85), and postpartum hemorrhage (0.50 ± 0.24 g/L, *P* = .16), respectively, but no significant differences were found. There was no significant difference in the Fg:C level between patients with (0.55 ± 0.03 g/L) and without postpartum thrombosis (0.47 ± 0.20 g/L, *P* = .42).

### 3.3 Genetic analysis

A total of 10 gene mutations were detected in 22 subjects from 8 unrelated pedigrees, including 1 homozygous missense mutation, 8 heterozygous missense mutations, and 1 splice mutation. Three compound missense mutations in the *FGA*, *FGB*, and *FGG* genes of 3 patients with congenital Hypodys- from the same family were identified. In this family, the sibling probands presented with moderate epistaxis, and their mother suffered postpartum hemorrhage after vaginal delivery for the first child. Furthermore, 1 girl with congenital Dys-, which was caused by 1 heterozygous missense mutation (c.991A>G) at the *FGG* exon 9, experienced menometrorrhagia (present since menarche and for more than 28 months) and menostaxis (prolonged lasting from 10 days to 1 month), whereas her mother was asymptomatic. Another heterozygous missense mutation (c.425T>G) at the *FGB* exon 3 was identified in 3 patients with congenital Hypo- from 1 family, including the probands with epistaxis, his asymptomatic elder brother, and his mother with bruises. Notably, the splice mutation c.180+1G>A at the junction of exon 2–intron 2 was first identified as a novel mutation in 4 patients with congenital Hypodys- from 1 family (HGMD, Clinvar database; https://site.geht.org/base-de-donnees-fibrinogene/). In this family, the probands, his uncle, and his grandfather presented with bleeding from minor wounds (for more than 10 minutes) in their daily life. His mother suffered postpartum hemorrhage after cesarean delivery for her second child. The results were shown in Table 2.

**Table 2:** Genotype and clinical phenotype in patients with congenital fibrinogen disorders (*N* = 22).

Mutation	Gene exon/intron	Protein mutation	Phenotype	Total (*n*)	Probands (*n*)	Relatives (*n*)	Type
g.1202C>T (Het)	*FGA* exon 2	Missense, Arg16Cys	Epistaxis, bruises, and menorrhagia	3	1	2	Dys-
c.104G>A (Het)	*FGA* exon 2	Missense, Arg35His	Menorrhagia; bleeding from minor wounds	3	1	2	Dys-
c.5678G>A (Het)	*FGG* exon 8	Missense, p.Arg275His	Asymptomatic	2	1	1	Dys-
g.7476G>A (Het)	*FGG* exon 8	Missense, p.Arg275His	Asymptomatic	2	1	1	Dys-
c.1178T>C (Het)	*FGG* exon 9	Missense, p.IIe367Thr	Epistaxis and menorrhagia; asymptomatic	2	1	1	Dys-
c.425T>G (Het)	*FGB* exon 3	Missense, p.Leu121Arg	Epistaxis, bruises, and asymptomatic	3	1	2	Hypo-
c.991A>G +c.510T>A +c.902G>A (CH)	*FGA* exon 5 *FGB* exon 4 *FGG* exon 8	Nonsense, p.Thr331Ala +Missense, p.Asn170Lys +Missense, p.Arg301His	Epistaxis Epistaxis Postpartum hemorrhage	3	1	2	Hypodys-
c.180+1G>A (Het)	*FGA* exon 2-intron 2 junction	Donor splice-site	Bleeding from minor wounds (*n* = 3); postpartum hemorrhage	4	1	3	Hypodys-

CH, compound heterozygous; Dys-, dysfibrinogenemia; Het, heterozygous; Hom, homozygous; Hypo-, hypofibrinogenemia; Hypodys-, hypodysfibrinogenemia.

### 3.4 Clinical characteristics of patients with 3 types of CFDs

According to the levels of Fg:C and Fg:Ag and the fibrinogen gene analysis, a total of 3 patients were diagnosed with congenital hypofibrinogenemia (family 17; Supplementary Table S1), 16 with congenital hypodysfibrinogenemia (families 6, 12, 22, 25, and 26; Supplementary Table S1), and 74 with congenital dysfibrinogenemia. Three patients with congenital dysfibrinogenemia had suffered from postpartum DVT, and no thrombosis occurred in the other 2 groups. Among the 53 patients with bleeding, at least 1 bleeding episode was identified in 3.8% (2/53), 20.8% (11/53), and 75.5% (40/53) patients with Hypo-, Hypodys-, and Dys-, respectively. The levels of Fg:C and Fg:Ag in patients with Hypodys- (0.32 ± 0.14 g/L; 1.55 ± 0.11 g/L) were lower than those in patients with Dys- (0.44 ± 0.19 g/L, *P* < .05; 2.22 ± 0.38 g/L, *P* < .0001). The Fg:C levels of bleeders were slightly lower in Hypodys- group (0.35 ± 0.14 g/L) than in Dys- group (0.42 ± 0.18 g/L; *P* > .05), whereas the overall ISTH-BAT bleeding scores of bleeders in Hypodys- group (median, 2; range, 0-7) were similar to those in Dys- group (median, 2; range, 0-7; *P* > .05). No significant differences were observed for other variables between the 2 groups. The results were shown in Table 3.

**Table 3:** Demographic and clinical characteristics of patients with 3 types of congenital fibrinogen disorders.

Parameters	Hypofibrinogenemia (*n* = 3)	Hypodysfibrinogenemia (*n* = 16)	Dysfibrinogenemia (*n* = 74)	*P* value
Age (y), median (IQR)	10 (7-38)	21 (8.4-38)	24.5 (8-37)	.85
Sex (male/female)	2/1	9/7	35/39	.52
Thrombosis, *n* (%)	0	0	3 (4.1)	.41
Bleeding episodes, *n* (%)	2 (66.7)	11 (68.8)	40 (54.1)	.28
ISTH-BAT bleeding score, median (IQR)	0 (0, 2)	1 (0-3)	1 (0-2.25)	.51
ISTH-BAT bleeding score of bleeders, median (IQR)	1 (0, 2)	2 (1-3)	2 (1-3)	.75
Bleeding score, *n* (%)				
0	2 (66.7)	6 (37.5)	35 (47.30)	
1	0 (0)	3 (18.75)	11 (14.86)	
2	1 (33.3)	2 (12.5)	10 (13.51)	
3	0 (0)	3 (18.75)	9 (12.16)	
4	0 (0)	1 (6.25)	5 (6.76)	
5	0 (0)	0 (0)	3 (4.05)	
7	0 (0)	1 (6.25)	1 (1.35)	
Laboratory characteristics (mean ± SD)				
PT (s)	12.10 ± 0.56	12.83 ± 0.89	12.63 ± 0.98	.47
aPTT (s)	28.27 ± 0.72	28.11 ± 2.78	28.41 ± 2.60	.67
TT (s)	24.3 ± 0.20	28.50 ± 2.87	29.51 ± 4.26	.36
Fg:C (g/L)	1.15 ± 0.11	0.32 ± 0.14	0.44 ± 0.20	.02^a^
Fg:Ag (g/L)	1.47 ± 0.09	1.55 ± 0.11	2.22 ± 0.38	<.0001^b^
Fg:C/Fg:Ag	0.78 ± 0.09	0.21 ± 0.09	0.20 ± 0.09	.82
Fg:Ag/Fg:C	1.29 ± 0.15	5.87 ± 3.06	6.06 ± 3.11	.81
Fg:C (g/L) of bleeders	1.13 ± 0.15	0.35 ± 0.14	0.42 ± 0.18	.20

Age and bleeding score of patients with Hypo- group are presented as median (the minimum values-the maximum values).

aPTT, activated partial thromboplastin time; Fg:Ag, fibrinogen antigen; Fg:C, fibrinogen activity; ISTH-BAT, International Society on Thrombosis and Haemostasis bleeding assessment tool; PT, prothrombin time; TT, thrombin time.

a *P* < .05.

b *P* < .0001 (hypodysfibrinogenemia vs dysfibrinogenemia groups, due to a small number of cases of patients with hypofibrinogenemia).

### 3.5 Clinical management

In fact, 65 (69.9%) subjects with no or mild bleeding symptoms did not receive any replacement therapy. Twenty-two (21.5%) subjects had no bleeding after surgery without preoperative replacement therapy, including circumcision (*n* = 7), eyelid mass excision (*n* = 3), excision of skin abscess (*n* = 2), frenectomy (*n* = 1), excision of hydrocele of testis (*n* = 1), excision of mole (*n* = 1), tooth extraction (*n* = 1), and cesarean delivery (*n* = 6). Nine (9.7%) pediatric patients had no bleeding after surgery who received preoperative 1 to 5 fibrinogen infusions (a daily dosage of 25-40 mg/kg), including circumcision (*n* = 4), adenotonsillectomy (*n* = 1), indirect inguinal hernia hernioplasty (*n* = 1), hemangiomatectomy of lower limb (*n* = 1), aspiration of hematoma of scalp (*n* = 1), and gastroscopy (*n* = 1; Table 4). Three (3.2%) subjects with bleeding after tooth extraction received packing and or suture treatment. One (1.1%) subject with bleeding after mammary fibroma surgery received 500 mL of fresh frozen plasma (FFP) transfusion. Additionally, for the patient with a history of 2 spontaneous abortions, from the beginning of third pregnancy until delivery, antenatal management included a combination of fibrinogen infusions (40 mg/kg) to maintain fibrinogen plasma concentrations (Clauss) above 1 g/L and anticoagulant therapy. She eventually had a full-term vaginal delivery. For the prophylaxis of thrombotic episodes, low-molecular-weight heparin at a dosage of 40 to 60 IE/kg was administered postpartum for 2 weeks. Five (5.4%) subjects with postpartum hemorrhage received 500- to 800-mL FFP transfusion (2 vaginal births and 3 cesarean deliveries). Two (2.2%) children with epistaxis received 1 to 2 fibrinogen infusions, with a daily dosage of 30 to 40 mg/kg. Thirty (3.2%) cases of menorrhagia received intermittent hormone therapy, and 5 (5.4%) cases of menorrhagia received intermittent iron therapy. Furthermore, 1 (1.1%) subject had severe active bleeding after adenotonsillectomy, with 1 preoperative fibrinogen infusion (30 mg/kg), and postoperative bleeding was controlled after 10 fibrinogen infusions (a daily dosage of 30 mg/kg). Twelve (12.9%) patients receiving fibrinogen infusion (a daily dosage of 25-40 mg/kg) had good outcomes, with the mean value of fibrinogen level of 1.05 g/L (range, 0.85-1.37 g/L) after infusion.

**Table 4:** Type of operation, postoperative bleeding, and treatment of 39 patients with congenital fibrinogen disorders.

Type of operation	Total (*n*)	Preoperative replacement therapy		Bleeding after surgery (*n*)	Postoperative replacement therapy	
		*n*	Treatment		*n*	Treatment
Highly fibrinolytic activity site						
Tooth extraction	4	0		4	3	Packing (*n* = 1) or suture (*n* = 2)
Adenotonsillectomy	2	2	Fg infusion (30-40 mg/kg)	1	1	Fg infusion (30 mg/kg)
Frenectomy	1	0		0	0	
Nonhighly fibrinolytic activity site						
Excision of skin abscess	2	0		0	0	
Eyelid mass excision	3	0		0	0	
Excision of mammary fibroma	1	1	400 mL FFP	0	0	
Excision of mole	1	0		0	0	
Circumcision	11	4	Fg infusion (30-40 mg/kg)	0	0	
Excision of hydrocele of testis	1	0		0	0	
Indirect inguinal hernia hernioplasty	1	1	200 mL FFP + Fg infusion (30 mg/kg)	0	0	
Hemangiomatectomy of lower limb	1	1	Fg infusion (40 mg/kg)	0	0	
Cesarean delivery	11	0	400-800 mL FFP	3	3	500-800 mL FFP
Aspiration of hematoma of scalp	1	1	Fg infusion (30 mg/kg)	0	0	
Gastroscopy	1	1	Fg infusion (25 mg/kg)	0	0	

FFP, fresh frozen plasma; Fg, fibrinogen.

Fg:C levels were slightly lower in patients with bleeding after surgery (0.36 ± 0.1 g/L) than in those without bleeding (0.44 ± 0.22 g/L, *P* > .05) after surgery, whereas the ISTH-BAT bleeding scores in patients with bleeding after surgery (median, 3; range, 1-4) were higher than in those without bleeding (median, 1; range, 0-5; *P* < .05). There was no significant difference in the incidence of postoperative hemorrhage between highly and nonhighly fibrinolytic activity sites for patients with prophylactic treatment (*P* = .12), whereas for patients without prophylactic treatment, the incidence of postoperative hemorrhage at highly fibrinolytic activity sites (80%) was higher than that at nonhighly fibrinolytic activity sites (12%, *P* < .05). Overall, Fg:C levels were lower in patients who needed treatment (0.33 ± 0.14 g/L) than in those who did not (0.48 ± 0.25 g/L, *P* < .01), with the optimal Fg:C cutoff level of 0.44 g/L.

Notably, 1 girl with congenital Dys-, which was caused by 1 heterozygous missense mutation (c.991A>G) at the *FGG* exon 9, received hemostatic drug (tranexamic acid tablets) and treatment with 25 fibrinogen infusions (1 infusion per month with a dose of 30 mg/kg). The menstrual period became shorter, but she still presents menometrorrhagia. Finally, after multidisciplinary (pediatric gynecology, pediatric hematology department, and clinical laboratory department) consultation, the girl received intrauterine device therapy to control the bleeding. The clinical effect of all 93 patients was considered very good in all events.

## 4 Discussion

We have reported a retrospective analysis of 93 subjects with CFDs identified from 36 unrelated families. Currently, the laboratory diagnosis and classification of CFDs rely on detection of fibrinogen activity and antigen and then genetic evaluation [12,16,18,19]. The 93 subjects exhibited prolonged TT, significantly decreased Fg:C, and normal or decreased Fg:Ag, which aligns with the previously proposed laboratory diagnosis for CFDs [14,16].

In our study, the main clinical association of CFDs was bleeding, which was identified in 57% subjects. Nevertheless, we recorded bleeding symptoms if they fulfilled the criteria for a bleeding score of ≥1 in the consensus ISTH-BAT [8,9], thus capturing mild bleeding episodes. Most subjects had bleeding at a single anatomical site, and only 24.7% of subjects had a bleeding score of ≥3. These findings suggest that most subjects with CFDs experienced mild bleeding, which is consistent with previous studies [[20], [21], [22]]. Meanwhile, we found that the severity of bleeding symptoms was negatively correlated with the level of Fg:C (*P* < .0001), which is consistent with previous reports [20,23]. Thrombosis was identified in 3.2% subjects in our study. Follow-up studies should be regularly conducted in patients with CFDs to record new bleeding or thrombotic events.

Herein, females were more prone to experience bleeding (*P* < .0001). We found that 23/47 (48.9%) females had suffered from gynecologic and pregnancy complications, including menorrhagia and/or prolonged menostaxis, miscarriage, postpartum hemorrhage, and postpartum thrombosis. Pregnancy is a high-risk clinical situation in women who have CFDs [24], although most pregnancies progress smoothly. Therefore, pregnancy should be managed by a multidisciplinary team consisting of hematologists, obstetricians, anesthetists, and clinical laboratory technician.

Our study reported 3 patients with hypodysfibrinogenemia from the same family who had 3 compound missense mutations in the *FGA*, *FGB*, and *FGG* genes. One of the mutations, c.991A>G (p.Thr331Ala) of *FGA* gene, was a known single nucleotide polymorphism without pathogenicity (https://www.ncbi.nlm.nih.gov/snp/rs6050). The other 2 mutations, c.510T>A (p.Asn170Lys) in the exon 4 of *FGB* [25] gene and c.902G>A (Arg301His) in the exon 8 of *FGG* gene, were identified as pathogenic mutations [21]. Interestingly, the mutation c.510T>A (p.Asn170Lys) in *FGB* exon 4 was first identified in Chinese patients with congenital hypodysfibrinogenemia, which has been shown to affect the quality and quantity of the fibrinogen [25]. Notably, a novel splice mutation, c.180+1G>A, at the boundary of intron 2–exon 2 of *FGA* was first detected in 4 Hypodys- patients with mild-to-moderate bleeding from the same family. We found that the *FGA* gene expression in splice mutation group was significantly lower than that in wild-type group (*P* < .05). Even though the number of cases of CFDs is already quite substantial in the study, only a small number of families underwent fibrinogen gene analysis, and more mutations will be identified in the future.

Currently, there is no standard or consensus on the treatments of CFDs. Replacement products in many countries are used for the treatment or prevention of bleeding in CFDs, including FFP, cryoprecipitates, or human fibrinogen concentrate [14,26,27]. In our study, patients who needed treatment have lower Fg:C levels (*P* < .01) compared with those who did not. We recommended that prophylactic fibrinogen infusion should be properly considered for patients with Fg:C ≤0.44 g/L and for patients undergoing surgery at highly fibrinolytic activity sites. Fg:C levels that reached 1.05 g/L after infusion were efficacious and safe for on-demand treatment of bleeding and surgical prophylaxis. Because a small number of patients received fibrinogen infusions in the study, further clinical studies are needed to optimize the treatment guidelines for patients with CFDs.

## 5 Conclusions

Most patients with CFDs have mild or no bleeding symptoms. Females with CFDs may face gynecologic and pregnancy complications. Fibrinogen activity combined with fibrinogen antigen and pedigree investigation can improve the feasibility and accuracy of diagnosis of CFDs. The severity of bleeding symptoms was negatively correlated with the levels of Fg:C.
