# Supplementary material for: Clinical phenotype and laboratory characteristics of 93 patients with congenital fibrinogen disorders from unrelated 36 families
Source: Res Pract Thromb Haemost. 2024 May 17;8(4):102445. doi: 10.1016/j.rpth.2024.102445 (PMC11215109; doi:10.1016/j.rpth.2024.102445)
Supplement: Supplementary material [file mmc1.doc]

Table S1 Demographic data, clinical phenotype, laboratory characteristics, genetic data, and type of 93 patients with congenital fibrinogen disorders from unrelated 36 families

| Family | Case | Sex | Age  (Y) | PT  (s) | APTT (s) | TT  (s) | Fg:C (g/L)  (Clauss) | Fg:C  (g/L )  (PT-der) | Fg:Ag  /Fg:C | Fg:C  /Fg:Ag | First  diagnosis | Clinical symptoms | Medical attention or treatment | BAT Bleed-ing scores | Gene analysis | Type |  |
| --- | --- | --- | --- | --- | --- | --- | --- | --- | --- | --- | --- | --- | --- | --- | --- | --- | --- |
| 1 | 1 III_1_ | M | 0.58 | 14.0 | 26.4 | 40.4 | 0.17 | 2.16 | 12.71 | 0.08 | Frenectomy | Bruises | Extensive | 3 | ND | Dys- |  |
|  | 2 II_1_ | M | 26 | 12.0 | 27.9 | 26.3 | 0.46 | 2.18 | 4.74 | 0.21 |  | A | Untreated | 0 | ND | Dys- |  |
| 2 | 3 III1 | F | 12.08 | 14.4 | 29.6 | 41.1 | 0.2 | 2.13 | 10.65 | 0.09 | Epistaxis | Epistaxis, Menorrhagia | Packing,  Fg infusions | 7 | ND | Dys- |  |
|  | 4 II2 | F | 38 | 13.5 | 30.3 | 35.5 | 0.16 | 2.05 | 12.81 | 0.08 |  | Menorrhagia | Consultation | 2 | ND | Dys- |  |
| 3 | 5 III1 | M | 8 | 14.5 | 34.1 | 32.6 | 0.11 | 2.21 | 20.09 | 0.05 | Circumcision | Epistaxis | Consultation | 2 | ND | Dys- |  |
|  | 6 II1 | M | 42 | 14.0 | 26.9 | 32 | 0.31 | 2.26 | 7.29 | 0.14 |  | A | Untreated | 0 | ND | Dys- |  |
| 4 | 7 III1 | F | 11 | 12.6 | 31.6 | 27.9 | 0.56 | 1.87 | 3.34 | 0.30 | Adenotonsillecto-my | A | Untreated | 0 | *FGG* exon 8  c.5678 G>A  p.Arg275His | Dys- |  |
|  | 8 II1 | M | 42 | 12.2 | 32.6 | 29.3 | 0.49 | 2.24 | 4.57 | 0.22 |  | A | Untreated | 0 | *FGG* exon 8  c.5678 G>A  p.Arg275His | Dys- |  |
| 5 | 9 III1 | F | 1.17 | 11.6 | 24.6 | 26.9 | 0.48 | 3.52 | 7.33 | 0.14 | Physical examination | Bruises | ≥5 bruises in exposed areas | 1 | ND | Dys- |  |
|  | 10 III2 | F | 12 | 13.5 | 31.6 | 26.8 | 0.3 | 2.12 | 7.07 | 0.14 |  | Menorrhagia | Iron therapy | 2 | ND | Dys- |  |
|  | 11 II1 | F | 37 | 13.2 | 27.0 | 27.7 | 0.26 | 1.89 | 7.27 | 0.14 |  | Menorrhagia | Hormonal therapy | 2 | ND | Dys- |  |
| 6 | 12 III1 | M | 6 | 11.5 | 27.8 | 28.3 | 0.58 | 1.66 | 2.86 | 0.35 | Circumcision | A | Untreated | 0 | ND | Hypodys- |  |
|  | 13 II1 | M | 35 | 11.2 | 27.1 | 27.9 | 0.40 | 1.56 | 3.9 | 0.26 |  | A | Untreated | 0 | ND | Hypodys- |  |
| 7 | 14 III1 | M | 9 | 14.3 | 32.6 | 27.1 | 0.63 | 2.88 | 4.57 | 0.22 | Circumcision | A | Untreated | 0 | ND | Dys- |  |
|  | 15 III2 | M | 16 | 12.8 | 26.7 | 33.7 | 0.35 | 2.04 | 5.83 | 0.17 |  | Bruises | ≥5 bruises in exposed areas | 1 | ND | Dys- |  |
|  | 16 II1 | F | 37 | 11.3 | 24.8 | 29.2 | 0.51 | 3.15 | 6.18 | 0.16 |  | Postpartum hemorrhage | 800 ml FFP | 3 | ND | Dys- |  |
| 8 | 17 III1 | M | 5.17 | 11.9 | 28.9 | 34.2 | 0.23 | 1.83 | 7.96 | 0.13 | Epistaxis | Epistaxis,  Bruises | Consultation,  Extensive | 5 | *FGA* exon 2  g.1202 C>T  Arg16Cys | Dys- |  |
|  | 18 II1 | F | 32 | 11.1 | 29.5 | 28.2 | 0.35 | 1.93 | 5.51 | 0.18 |  | Menorrhagia and menostaxis | Consultation | 1 | *FGA* exon 2  g.1202 C>T  Arg16Cys | Dys- |  |
|  | 19 I1 | F | 54 | 11.7 | 26.3 | 25.2 | 0.59 | 3.01 | 5.10 | 0.20 |  | Menorrhagia | Consultation | 1 | *FGA* exon 2  g.1202 C>T  Arg16Cys | Dys- |  |
| 9 | 20 III1 | F | 11 | 12.6 | 29.2 | 26.2 | 0.34 | 1.86 | 5.47 | 0.18 | Eyelid mass excision | Menorrhagia and menostaxis | IUD+Fg infusions+  Iron therapy | 4 | *FGG* exon 9  C.1178 T>C  p.IIe367Thr | Dys- |  |
|  | 21 II1 | F | 39 | 11.5 | 26.9 | 25.2 | 0.46 | 2.07 | 4.5 | 0.22 |  | A | Untreated | 0 | *FGG* exon 9  C.1178 T>C  p.IIe367Thr | Dys- |  |
| 10 | 22 III1 | M | 4.75 | 12.6 | 29.7 | 24.5 | 0.49 | 1.90 | 3.88 | 0.26 | Epistaxis | Epistaxis | Consultation | 2 | ND | Dys- |  |
|  | 23 II1 | F | 38 | 11.7 | 26.1 | 27.2 | 0.52 | 1.93 | 3.71 | 0.27 |  | Thrombosis | Anticoagulant therapy | 0 | ND | Dys- |  |
|  | 24 III2 | M | 10 | 12.9 | 27.4 | 28.5 | 0.51 | 2.10 | 4.12 | 0.24 |  | A | Untreated | 0 | ND | Dys- |  |
|  | 25 III3 | F | 13 | 11.9 | 29.1 | 28.2 | 0.41 | 2.18 | 5.32 | 0.19 |  | Epistaxis | >5 per year | 1 | ND | Dys- |  |
|  | 26 II3 | F | 36 | 12.4 | 30.5 | 28.3 | 0.58 | 2.56 | 4.41 | 0.23 |  | Thrombosis | Anticoagulant therapy | 0 | ND | Dys- |  |
| 11 | 27 III1 | M | 11 | 12.9 | 28.2 | 32.8 | 0.35 | 1.98 | 5.66 | 0.18 | Circumcision | A | Untreated | 0 | ND | Dys- |  |
|  | 28 II1 | F | 41 | 12.1 | 26.9 | 29.6 | 0.59 | 2.38 | 4.03 | 0.25 |  | Menorrhagia | Trivial | 0 | ND | Dys- |  |
| 12 | 29 III1 | F | 9 | 13.4 | 28.9 | 29.5 | 0.36 | 1.56 | 4.33 | 0.23 | Epistaxis | Epistaxis | Fg infusions | 4 | Three compound mutations | Hypodys- |  |
|  | 30 III2 | M | 7 | 13.8 | 26.6 | 30.4 | 0.27 | 1.27 | 4.70 | 0.21 | Epistaxis | Epistaxis | Consultation | 2 | Three compound mutations | Hypodys- |  |
|  | 31 II1 | F | 38 | 12.0 | 36.2 | 28.4 | 0.38 | 1.42 | 3.74 | 0.27 |  | Postpartum hemorrhage | 400 ml FFP | 3 | Three compound mutations | Hypodys- |  |
| 13 | 32 III1 | M | 5.75 | 12.4 | 28.2 | 25.8 | 0.32 | 2.35 | 7.34 | 0.14 | Circumcision | Epistaxis | Fg infusions | 4 | ND | Dys- |  |
|  | 33 II1 | F | 30 | 11.5 | 29.5 | 36.1 | 0.23 | 1.93 | 8.39 | 0.12 |  | Postpartum hemorrhage | 500 ml FFP | 3 | ND | Dys- |  |
| 14 | 34 III1 | M | 5.08 | 11.7 | 24.2 | 28.5 | 0.47 | 2.18 | 4.64 | 0.22 | Epistaxis | Epistaxis,  Bruises | Consultation,  Consultation | 3 | ND | Dys- |  |
|  | 35 III2 | F | 12 | 11.9 | 26.5 | 29.3 | 0.43 | 1.93 | 4.49 | 0.22 |  | Menorrhagia | Iron therapy | 2 | ND | Dys- |  |
|  | 36 II1 | F | 34 | 11.3 | 28.4 | 27.2 | 0.55 | 1.85 | 3.36 | 0.30 |  | Menorrhagia | Consultation | 1 | ND | Dys- |  |
| 15 | 37 III1 | M | 7 | 12.9 | 28.2 | 31.6 | 0.51 | 2.14 | 4.20 | 0.24 | Circumcision | A | Untreated | 0 | ND | Dys- |  |
|  | 38 II1 | F | 35 | 12.1 | 26.6 | 29.8 | 0.48 | 2.01 | 4.19 | 0.24 |  | A | Untreated | 0 | ND | Dys- |  |
|  | 39 II2 | M | 22 | 11.9 | 27.3 | 30.1 | 0.38 | 1.87 | 4.92 | 0.20 |  | Epistaxis, Gum bleeding | Packing, Consultation | 5 | ND | Dys- |  |
|  | 40 I1 | M | 61 | 11.3 | 27.1 | 32.8 | 0.41 | 1.98 | 4.83 | 0.21 |  | Epistaxis | >5 per year | 1 | ND | Dys- |  |
| 16 | 41 III1 | M | 6.67 | 12.5 | 30.9 | 24.9 | 0.75 | 1.85 | 2.47 | 0.41 | Circumcision | A | Untreated | 0 | ND | Dys- |  |
|  | 42 II1 | F | 38 | 13.5 | 29.7 | 24.8 | 0.67 | 2.04 | 3.04 | 0.33 |  | A | Untreated | 0 | ND | Dys- |  |
| 17 | 43 III1 | M | 7 | 12.2 | 27.8 | 24.1 | 1.02 | 1.46 | 1.43 | 0.70 | Epistaxis | Epistaxis | Consultation | 2 | *FGB* exon 3  c.425T>G p.Leu121Arg | Hypo- |  |
|  | 44 III1 | M | 10 | 12.6 | 29.1 | 24.3 | 1.20 | 1.56 | 1.30 | 0.77 |  | A | Untreated | 0 | *FGB* exon 3  c.425T>G p.Leu121Arg | Hypo- |  |
|  | 45 II1 | F | 38 | 11.5 | 27.9 | 24.5 | 1.23 | 1.39 | 1.13 | 0.88 |  | Bruises | Trivial | 0 | *FGB* exon 3  c.425T>G p.Leu121Arg | Hypo- |  |
| 18 | 46 III1 | M | 13 | 12.9 | 26.6 | 24.0 | 0.82 | 2.05 | 2.50 | 0.40 | Bleeding from minor wounds | Bleeding from minor wounds, Gastrointestinal bleeding | More than 10 min, Fg infusions | 5 | *FGA* exon 2  c.104G>A  Arg35His | Dys- |  |
|  | 47 II1 | F | 38 | 12.2 | 27.4 | 25.3 | 0.89 | 2.15 | 2.42 | 0.41 |  | Menorrhagia | Consultation | 1 | *FGA* exon 2  c.104G>A  Arg35His | Dys- |  |
|  | 48 I1 | F | 60 | 11.8 | 29.1 | 25.8 | 0.83 | 2.03 | 2.45 | 0.41 |  | Menorrhagia | Consultation | 1 | *FGA* exon 2  c.104G>A  Arg35His | Dys- |  |
| 19 | 49 III1 | F | 2.25 | 12.2 | 28.9 | 23.6 | 0.96 | 2.28 | 2.65 | 0.38 | Hemangiomatectomy of lower limb | A | Untreated | 0 | ND | Dys- |  |
|  | 50 II1 | M | 28 | 12.6 | 28.5 | 23.8 | 1.13 | 2.45 | 2.17 | 0.46 |  | A | Untreated | 0 | ND | Dys- |  |
| 20 | 51 III1 | M | 2.33 | 13.6 | 25.5 | 31.6 | 0.30 | 1.92 | 6.40 | 0.16 | Physical examination | A | Untreated | 0 | *FGG* exon 8  g.7476G>A  p.Arg275His | Dys- |  |
|  | 52 II1 | F | 27 | 12.3 | 27.8 | 28.7 | 0.55 | 2.41 | 4.38 | 0.23 |  | A | Untreated | 0 | *FGG* exon 8  g.7476G>A  p.Arg275His | Dys- |  |
| 21 | 53 III1 | F | 11 | 12.9 | 29.9 | 30.4 | 0.47 | 2.37 | 5.04 | 0.20 | Excision of skin abscess | A | Untreated | 0 | ND | Dys- |  |
|  | 54 II1 | M | 36 | 11.5 | 26.6 | 29.0 | 0.55 | 3.04 | 5.53 | 0.18 |  | A | Untreated | 0 | ND | Dys- |  |
| 22 | 55 III1 | M | 4.33 | 12.2 | 23.8 | 26.8 | 0.50 | 1.55 | 3.10 | 0.32 | Abdominal pain | Bleeding from minor wounds | Trivial | 0 | *FGA* exon 2-intron 2 junction, c.180+1G>A | Hypodys- |  |
|  | 56 II1 | F | 37 | 13.3 | 26.2 | 25.3 | 0.39 | 1.55 | 3.97 | 0.25 |  | Postpartum hemorrhage | 500 ml FFP | 3 | *FGA* exon 2-intron 2 junction, c.180+1G>A | Hypodys- |  |
|  | 57 II2 | M | 31 | 13.4 | 24.8 | 24.4 | 0.48 | 1.57 | 3.27 | 0.31 |  | Bleeding from minor wounds | more than 10 min | 1 | *FGA* exon 2-intron 2 junction, c.180+1G>A | Hypodys- |  |
|  | 58 I1 | M | 64 | 12.3 | 28.0 | 23.8 | 0.40 | 1.57 | 3.93 | 0.25 |  | Bleeding from minor wounds | more than 10 min | 1 | *FGA* exon 2-intron 2 junction, c.180+1G>A | Hypodys- |  |
| 23 | 59 III1 | M | 10 | 13.0 | 30.3 | 30.4 | 0.31 | 1.85 | 5.97 | 0.17 | Circumcision | A | Untreated | 0 | ND | Dys- |  |
|  | 60 II1 | M | 42 | 12.1 | 27.1 | 29.4 | 0.46 | 2.40 | 5.21 | 0.19 |  | A | Untreated | 0 | ND | Dys- |  |
|  | 61 I1 | F | 61 | 12.1 | 27.8 | 29.5 | 0.41 | 2.42 | 5.90 | 0.17 |  | Bleeding after tooth extraction | Packing | 3 | ND | Dys- |  |
| 24 | 62 III1 | F | 5.58 | 13.3 | 29.4 | 27.5 | 0.56 | 1.91 | 3.41 | 0.29 | Eyelid mass excision | A | Untreated | 0 | ND | Dys- |  |
|  | 63 II1 | F | 33 | 13.5 | 31.6 | 36.3 | 0.56 | 1.87 | 3.34 | 0.30 |  | A | Untreated | 0 | ND | Dys- |  |
| 25 | 64 III1 | F | 11 | 13.1 | 28.8 | 26.7 | 0.16 | 1.61 | 10.06 | 0.10 | Bruises | Bruises | Extensive | 3 | ND | Hypodys- |  |
|  | 65 III2 | M | 9 | 13.0 | 28.7 | 31.6 | 0.28 | 1.60 | 5.71 | 0.18 |  | A | Untreated | 0 | ND | Hypodys- |  |
|  | 66 II1 | M | 40 | 12.2 | 27.7 | 32.8 | 0.28 | 1.66 | 5.93 | 0.17 |  | A | Untreated | 0 | ND | Hypodys- |  |
|  | 67 I1 | M | 63 | 13.3 | 27.0 | 31.2 | 0.11 | 1.38 | 12.55 | 0.08 |  | Bleeding from minor wounds | Consultation | 2 | ND | Hypodys- |  |
| 26 | 68 III1 | F | 8.42 | 14.0 | 27.9 | 26.2 | 0.18 | 1.65 | 9.17 | 0.11 | Eyelid mass excision | Epistaxis | >5 per year | 1 | ND | Hypodys- |  |
|  | 69 III2 | F | 8.42 | 14.2 | 28.8 | 33.1 | 0.26 | 1.54 | 5.92 | 0.17 |  | A | Untreated | 0 | ND | Hypodys- |  |
|  | 70 II1 | F | 38 | 12.3 | 31.4 | 29.6 | 0.15 | 1.62 | 10.80 | 0.09 |  | Postpartum hemorrhage, Bleeding after mammary fibroma surgery | 800 ml FFP,  400 ml FFP | 7 | ND | Hypodys- |  |
| 27 | 71 III1 | M | 7.67 | 14.0 | 34.1 | 29.4 | 0.32 | 1.87 | 5.84 | 0.17 | Circumcision | A | Untreated | 0 | ND | Dys- |  |
|  | 72 II1 | M | 32 | 12.1 | 27.9 | 29.8 | 0.36 | 1.98 | 5.50 | 0.18 |  | Bleeding after tooth extraction, Gum bleeding | Packing and suture,  present | 4 | ND | Dys- |  |
|  | 73 I1 | F | 60 | 12.0 | 26.3 | 29.0 | 0.37 | 2.16 | 5.84 | 0.17 |  | Bleeding after tooth extraction | Packing and suture | 3 | ND | Dys- |  |
| 28 | 74 III1 | M | 4.75 | 16.0 | 30.4 | 33.7 | 0.20 | 2.53 | 12.65 | 0.08 | Physical examination | Bleeding from minor wounds, Bruises | More than10 min, Consultation | 3 | ND | Dys- |  |
|  | 75 II1 | F | 33 | 13.6 | 30.4 | 46.4 | 0.20 | 2.48 | 12.40 | 0.08 |  | Menorrhagia, Bruises | Iron therapy,  >1 cm in exposed areas | 3 | ND | Dys- |  |
|  | 76 I1 | F | 68 | 13.5 | 29.7 | 24.8 | 0.47 | 2.54 | 5.40 | 0.19 |  | Epistaxis | Consultation | 2 | ND | Dys- |  |
| 29 | 77 III1 | M | 4.08 | 12.7 | 21.7 | 29.3 | 0.45 | 2.01 | 4.47 | 0.22 | Excision of hydrocele of testis | A | Untreated | 0 | ND | Dys- |  |
|  | 78 II1 | F | 38.42 | 13.2 | 30.0 | 36.3 | 0.19 | 2.18 | 11.47 | 0.09 |  | Menorrhagia and menostaxis | Hormonal therapy | 2 | ND | Dys- |  |
| 30 | 79 III1 | F | 5.08 | 10.6 | 27.4 | 25.2 | 0.73 | 2.08 | 2.85 | 0.35 | Physical examination | A | Untreated | 0 | ND | Dys- |  |
|  | 80 II1 | M | 29 | 11.5 | 21.0 | 25.0 | 0.79 | 2.12 | 2.68 | 0.37 |  | A | Untreated | 0 | ND | Dys- |  |
| 31 | 81 III1 | M | 0.33 | 13.5 | 36.1 | 27.9 | 0.25 | 2.19 | 8.76 | 0.11 | Aspiration of hematoma of scalp | Spontaneous hematoma | Fg infusions | 2 | ND | Dys- |  |
|  | 82 II1 | F | 23 | 13.3 | 32.6 | 37.5 | 0.25 | 2.18 | 8.72 | 0.11 |  | Bleeding from minor wounds | Consultation | 2 | ND | Dys- |  |
| 32 | 83 III1 | M | 13 | 12.8 | 27.8 | 28.1 | 0.21 | 2.43 | 11.57 | 0.09 | Adenotonsillectomy | Bleeding after adenotonsillect-omy | Fg infusions | 4 | ND | Dys- |  |
|  | 84 II1 | M | 37 | 13.7 | 29.7 | 30.8 | 0.24 | 2.29 | 9.54 | 0.10 |  | Bleeding after tooth extraction | No intervention | 1 | ND | Dys- |  |
| 33 | 85 III1 | F | 7.83 | 13.1 | 28.6 | 31.8 | 0.23 | 1.86 | 8.09 | 0.12 | Indirect inguinal hernia hernioplasty | Gastrointestinal bleeding | Iron therapy +Fg infusions | 4 | ND | Dys- |  |
|  | 86 II1 | F | 32 | 12.5 | 29.7 | 31.5 | 0.31 | 2.13 | 6.87 | 0.15 |  | A | Untreated | 0 | ND | Dys- |  |
|  | 87 I1 | M | 55 | 13.2 | 28.8 | 28.1 | 0.52 | 2.03 | 3.90 | 0.26 |  | A | Untreated | 0 | ND | Dys- |  |
| 34 | 88 III1 | M | 8 | 13.2 | 27.8 | 30.9 | 0.24 | 2.02 | 8.42 | 0.12 | Circumcision | Bruises | Extensive | 3 | ND | Dys- |  |
|  | 89 II1 | M | 35 | 12.9 | 27.2 | 28.3 | 0.36 | 2.47 | 6.86 | 0.15 |  | A | Untreated | 0 | ND | Dys- |  |
| 35 | 90 III1 | F | 10.67 | 12.1 | 25.9 | 28.8 | 0.36 | 1.87 | 5.19 | 0.19 | Pneumonia | A | Untreated | 0 | ND | Dys- |  |
|  | 91 II1 | F | 36 | 10.4 | 23.6 | 30.8 | 0.35 | 2.37 | 6.77 | 0.15 |  | Bleeding from minor wounds | More than 10 min | 1 | ND | Dys- |  |
| 36 | 92 III1 | M | 11 | 13.5 | 27.2 | 24.3 | 0.63 | 3.53 | 5.60 | 0.18 | Circumcision | A | Untreated | 0 | ND | Dys- |  |
|  | 93 II1 | F | 36 | 13.2 | 30.0 | 25.4 | 0.56 | 3.17 | 5.66 | 0.18 |  | Thrombosis | Anticoagulant therapy | 0 | ND | Dys- |  |

Abbreviations: I1, The proband’s grandfather or grandma; II1, The proband’s father or mother; II2, The proband's uncle or aunt; III1, Proband; III2 and III3, Brother or sister of the proband; Y, years; PT, prothrombin time; APTT, activated partial thromboplastin time; TT, thrombin time; Fg:C, fibrinogen activity; Fg:Ag, fibrinogen antigen; M, male; F, female; First diagnosis, 37 child probands were first identified during coagulation screening or bleeding; A, asymptomatic; Clinical symptoms, Historical symptoms of bleeding and thrombosis were determined by patient interview and inspection of clinical records; FFP, Fresh Frozen Plasma; BAT bleeding scores, International Society on Thrombosis and Haemostasis bleeding assessment tool; ND, not detect; Three compound mutations, FGA exon 5, c.991A>G, p.Thr331Ala + FGB exon 4, c.510T>A , p.Asn170Lys + FGG exon 8, c.902G>A, p.Arg301His; Hypodys-, Hypodysfibrinogenemia;Dys-, Dysfibrinogenemia; Hypo-, Hypofibrinogenemia.
